# Tibial Nerve Palsy Secondary to Spontaneous Isolated Popliteus Muscle Rupture and Localized Compartment Syndrome

**DOI:** 10.3390/diagnostics15232990

**Published:** 2025-11-25

**Authors:** Sophie Jolliet, Yves Harder, Sébastien Durand

**Affiliations:** 1 Department of Plastic, Reconstructive and Aesthetic Surgery and Hand Surgery, Centre Hospitalier Universitaire Vaudois (CHUV), 1011 Lausanne, Switzerland; sophie.jolliet@chuv.ch (S.J.); yves.harder@chuv.ch (Y.H.); 2 Faculty of Biology and Medicine, University of Lausanne, 1015 Lausanne, Switzerland

**Keywords:** compartment syndrome, popliteus muscle, soleus muscle, spontaneous muscle rupture, tibial nerve, tibial nerve palsy

## Abstract

We report the case of a 68-year-old man who developed sudden pain at the right calf, followed by progressive tibial nerve palsy. Magnetic resonance imaging (MRI) demonstrated localized signal abnormalities and swelling of the popliteus muscle compressing the tibial nerve. A medial surgical approach in supine position provided direct access to both the popliteus muscle and the tibial nerve while minimizing operative risk. Intraoperative findings confirmed necrosis of the popliteus muscle, which was subsequently supported by histopathology. Surgical decompression consisted of debridement of the necrotic tissue, associated with the release of the tibial nerve at the soleus arch. This case highlights a dual mechanism of tibial nerve compression: 1. swelling of the popliteus muscle following spontaneous rupture exerting a direct mass effect and 2. concomitant localized compartment syndrome. This dual mechanism led to significant transient tibial nerve palsy, which was successfully reversed following surgical decompression, with sustained recovery at ten months.

## 1. Introduction

**Figure 1:**
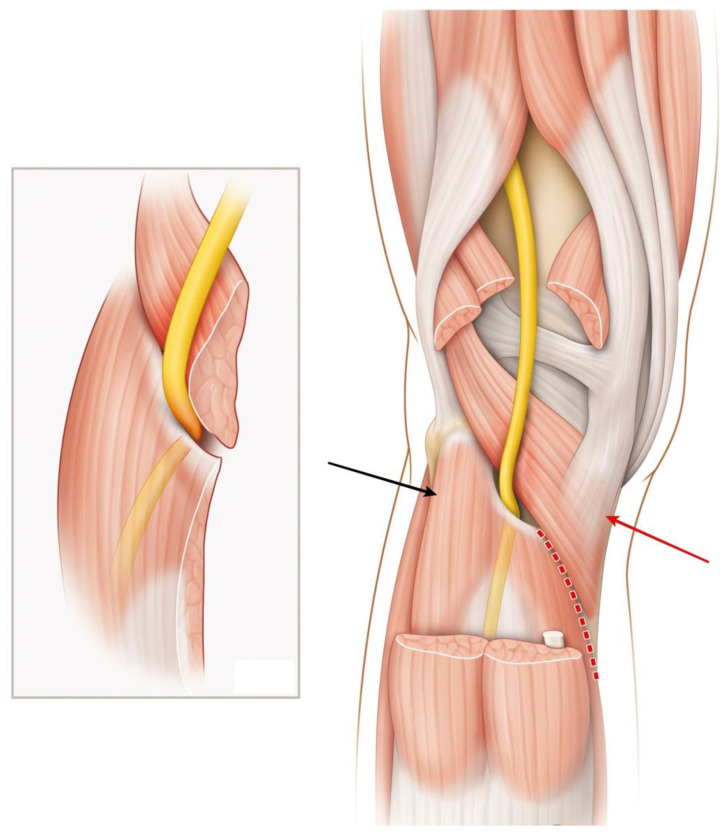
Posterior view of the popliteal fossa (both medial and lateral heads of gastrocnemius muscle, as well as the plantaris muscle, are removed) showing the tibial nerve (yellow) running over the popliteus muscle and beneath the soleal arch. Inset: mechanism of nerve entrapment during popliteus swelling. Black arrow: soleus muscle. Red arrow: popliteus muscle with semi-membranous expansion. Dotted line: medial surgical approach for tibial nerve release.

The popliteus muscle is a small triangular muscle lying on the floor of the popliteal fossa, extending obliquely from its proximal origin at the lateral femoral condyle to its distal insertion at the posterior tibia, as well as the posterior capsule of the knee, the lateral meniscus, and the fibular head [1]. The direct expansion of the semi-membranous muscle’s insertion blends into its medial aponeurosis. The popliteus muscle participates in knee flexion, rotates the tibia medially relative to the femur, and contributes to posteromedial knee stability [2]. At the level of the knee joint, the tibial nerve runs closely to the popliteal artery and its vein. The nerve lies posterior to the popliteus muscle and anterior to the tendinous arch of the soleus muscle, a well-defined and constant anatomical structure that makes it vulnerable to compression, in case of swelling of the popliteus muscle (Figure 1). Distal to the popliteus muscle, the tibial nerve innervates the soleus, the tibialis posterior (TP), the flexor digitorum longus (FDL), and the flexor hallucis longus (FHL) muscle, as well as all intrinsic muscles of the foot, and provides sensory supply to the heel and the sole of the foot [2].

Injuries to the popliteus generally occur in the setting of severe trauma and are often associated with ligamentous damages producing posterolateral knee instability, though clinically significant injuries of this muscle are rare. In a large MRI series [3], involvement of the popliteus muscle was identified in approximately 1% of the knee examinations and the rupture involved mainly its muscular portion in the large majority of the cases. Isolated injuries of the popliteus muscle are even less common [4].

MRI features of popliteus muscle injury include muscle enlargement and increased signal intensity on T2-weighted images [5].

A muscle rupture may result not only in the swelling of the affected muscle but also in the development of compartment syndrome [6], which can be limited to a single compartment or, in rare cases, only to an individual muscle [7].

We report a case of tibial nerve palsy secondary to spontaneous isolated popliteus muscle rupture, associated with compartment syndrome, and an alternative to the classical posterior surgical approach for selective nerve release. Furthermore, a specific review of the literature was undertaken.

## 2. Case Report

**Figure 2:**
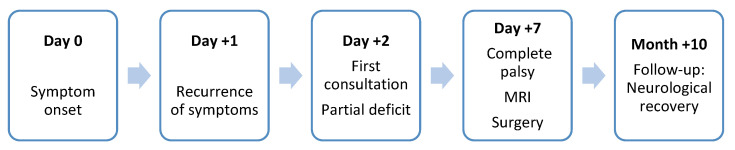
Visual clinical timeline summarizing the sequence of symptoms, diagnostic steps, and management.

A 68-year-old man with type II diabetes, obesity, and arterial hypertension experienced sudden, severe pain in his left popliteal fossa, while simply rising from his chair, without any trauma. Symptoms recurred 24 h later with increased pain, progressive anesthesia of the foot’s sole, and an inability to flex the ankle joint and the toes (Figure 2).

**Figure 3:**
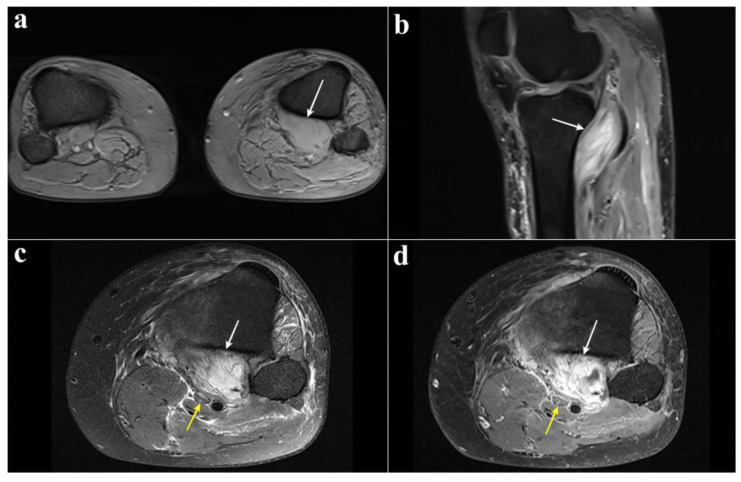
Axial T2-weighted MR images at the level of the proximal leg, comparing both right and left legs. There is a marked asymmetry of the popliteus muscles, with the left popliteus muscle (white arrow) appearing enlarged compared to the contralateral side on the right (**a**). Sagittal fat-suppressed proton density-weighted MR image of the left knee. The swollen popliteus muscle is visible (white arrow), exerting a mass effect with displacement of the popliteal vessels and the tibial nerve (**b**). Axial MR images of the proximal left leg. Fat-suppressed proton density-weighted sequence (**c**) and contrast-enhanced fat-suppressed T1-weighted sequence (**d**) demonstrating marked swelling and abnormal signal with diffuse enhancement of the popliteus muscle (white arrow) with posterior displacement of the tibial nerve (yellow arrow).

**Figure 4:**
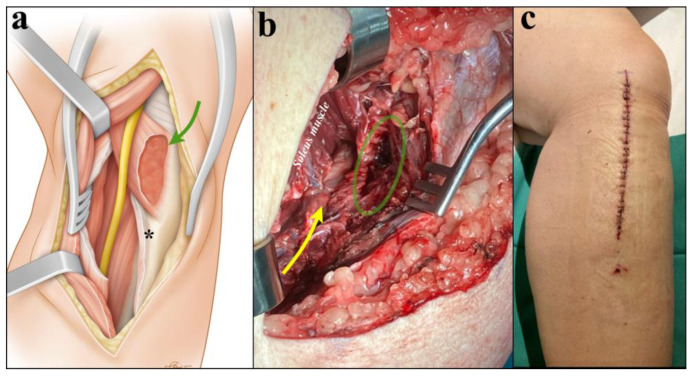
Schematic drawing showing surgical exposure through a longitudinal medial skin incision, showing the popliteus muscle (green arrow) after the opening of its overlying fascia, the soleus muscle, and the gastrocnemius muscle posteriorly (**a**). The soleus is detached from its tibial insertion (asterisk), allowing for a release of the tibial nerve (yellow). Intraoperative view (**b**) showing excision of the necrotic portion of the popliteus muscle (green circle). The soleus muscle is detached from its tibial insertion distally, allowing for the exposure and release of the tibial nerve (yellow arrow). (**c**) Postoperative scar located along the posteromedial border of the tibia. This medial approach facilitates postoperative wound monitoring and care, while minimizing the risk of maceration, when compared to a posterior approach.

MRI revealed edema and enlargement of the left popliteus muscle with increased signal intensity on T2-weighed images, demonstrating neurovascular compression (Figure 3). An electromyography was performed but was not interpretable due to marked edema of the left lower limb. Duplex sonography confirmed popliteal vein thrombosis. On admission, examination showed complete anesthesia of the left sole of the foot, which was associated with complete functional deficit (M0) of the FHL, FDL, and TP muscles. The left calf was swollen and asymmetrical. A longitudinal approach medial at the proximal third of the leg was performed, and the medial head of the gastrocnemius, the soleus, and the popliteus muscles were exposed. Despite the rupture observed on MRI, the popliteus muscle was still enclosed within an intact muscle compartment that was well delimited by a resistant fascia. The popliteus muscle compartment was firm on palpation and clear fluid drained after surgical incision of the compartment. Areas of the popliteus muscle showed signs of necrosis that were debrided radically. The soleus muscle was then detached from its medial insertion at the tibia and from the soleal line posteriorly, allowing for release of the soleal tendinous arch and eventually full decompression of the tibial nerve (Figure 4).

**Figure 5:**
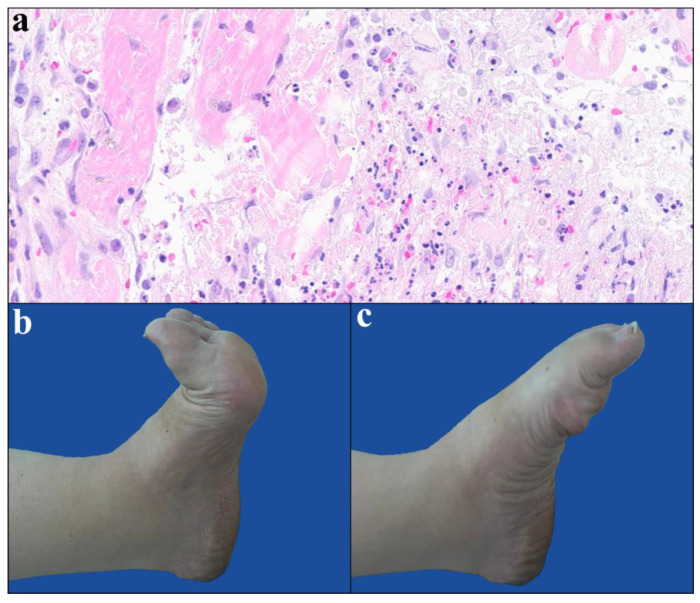
Hematoxylin and eosin (H&E) stained section of the popliteal muscle (magnification ×40). The section shows predominantly necrotic muscle fibers with marked infiltration of neutrophilic polymorphonuclear cells (**a**). Medial view of the left foot at 10 months after surgery; full extension (**b**). Full plantar flexion, illustrating complete functional recovery (**c**).

Histology confirmed muscle necrosis with both hemorrhagic and inflammatory changes (Figure 5).

Anticoagulation was initiated with low-molecular-weight heparin s.c. and subsequently switched to Rivaroxaban (Xarelto^®^) p.o. for a total duration of three months. At the end of this period, follow-up duplex ultrasound demonstrated complete recanalization of the popliteal vein.

At ten months follow-up, the patient was able to walk normally without any functional limitation, including toe flexion (M4+) and ankle flexion (M5). Plantar and toe sensation were restored (Figure 5).

## 3. Discussion

Tarsal tunnel syndrome is a relatively common neuropathy following entrapment of the tibial nerve. In contrast, high tibial nerve compression at the level of the popliteal fossa represents a rare site of entrapment [8]. Various causes have been described, including hypertrophy of the gastrocnemius tendon [9], the presence of an abnormal fibrous band between the two heads of the gastrocnemius muscle [10], fibers originating from the medial head of the gastrocnemius muscle, or compression by extra-neural mass lesions, such as a Baker cyst. Similarly, tibial nerve palsy related to popliteus muscle injury is rare and likely underreported. When evaluating pain or neurological symptoms arising in the popliteal fossa, several alternative diagnoses should also be considered. Deep venous thrombosis may present with acute calf pain and swelling and must be promptly ruled out. Rupture of the soleus or medial gastrocnemius muscle (tennis leg) can mimic both the clinical presentation and the biphasic pattern described in our patient. Other potential causes include popliteal cyst rupture, intramuscular hematoma, ganglion, iatrogenic nerve injuries, and, more rarely, nerve sheath tumor or popliteal artery aneurysms. These conditions can all produce mass effect and secondarily compress the tibial nerve, underscoring the importance of an appropriate differential diagnosis. A review of the literature (Table 1) identified only eight published cases [2,4,5,11,12,13,14,15], mostly affecting middle-aged patients (mean age: 52.3 years; two women and six men). In most cases, onset of symptoms was associated with minor trauma, such as twisting or pivoting of the knee joint, jumping, or specific sport-related activities, including tennis or running [2,4,12,14,15]. However, in at least two cases [5,13], including the herein presented case, no identifiable trauma was reported, suggesting that a spontaneous mechanism of an intact or degenerated popliteus muscle may also be involved.

Clinically, patients usually present with significant pain at the calf, followed by neurological symptoms ranging from decreased sensibility of the plantar area of the foot to complete palsy of the flexors of the toe (FHL or FDL). These reports often lack consistency and precision in the description of neurological deficits. Notably, muscle strength is not consistently documented using the standardized Medical Research Council (MRC) grading system, which is a practice that would greatly enhance comparability across cases. A noteworthy feature is the biphasic course observed in five of the eight cases [4,11,13,14,15], as seen in this patient. That is, they typically report a sudden onset of intense pain at the calf, which decreases rapidly. However, within 24 h, the pain reappears with an even greater intensity that is accompanied by progressive neurological deterioration. This clinical pattern is probably related to the initial muscle rupture, followed by subsequent compression of the tibial nerve following hematoma or muscle swelling within the compartment, and eventually becoming associated with compartment syndrome of the popliteus muscle.

MRI consistently revealed isolated abnormalities of the popliteus muscle, such as thickening due to edema, hematoma, or rupture creating a mass effect that compresses the tibial nerve and the popliteal vessels against the soleus arch. Five of the eight reported patients underwent conservative treatment [2,4,12,14,15]. Unfortunately, the long-term outcomes showed only partial neurological recovery in three cases, with persistent combined sensorimotor deficit, whereas two cases did not have follow-up documentation. Of the three surgically treated patients [5,11,13], pain resolved immediately after surgery. However, follow-up revealed persistent partial deficit in two cases, whereas one patient achieved an almost complete recovery. However, the reports did not document any interval between onset of symptoms and surgical treatment. However, the herein presented cases were decompressed surgically 7 days after symptom onset. Despite this time delay, the patient experienced almost complete neurological recovery, suggesting that timely intervention may help prevent permanent deficits. Indeed, we believe that prolongation of this dual mechanism may ultimately result in irreversible nerve injury. The limited number of reported cases make it impossible to draw a definitive conclusion, but this experience suggests that early surgery may yield a favorable outcome, particularly when symptoms progress or fail to improve spontaneously.

Iida et al. [5] show histological analysis of the popliteal muscle, revealing granulation tissue composed of striated muscle, connective tissue, proliferated vessels, and discrete lymphocyte infiltration of the popliteal muscle. This report represents the only documented case providing histopathological confirmation of popliteus muscle necrosis, though it should be mentioned that Bollier et al. [11] macroscopically observed the presence of necrosis of the popliteus muscle during surgery.

The neurological impairment observed in our patient can be reasonably explained by a dual mechanism combining direct mechanical and secondary ischemic injury. The acute enlargement of the ruptured popliteus muscle, often associated with hematoma and inflammatory edema, may induce focal compression of the tibial nerve within the popliteus muscle compartment. Because this compartment is relatively closed and poorly compliant, even modest increases in volume can significantly raise intracompartmental pressure, contributing to a secondary ischemic insult and increasing compression of the tibial nerve.

Another distinctive feature of this case is the use of a medial approach to access both the popliteus muscle and the tibial nerve. This approach provides several advantages compared to the classical posterior approach. First, it offers a rather direct access to the medial aspect of the popliteus muscle, without the need of dissection through multiple muscle layers. Second, it allows safer and more complete excision of necrotic tissue, which is eased in case a swollen popliteus muscle becomes more prominent beneath the medial surface of the tibia. Third, this approach facilitates postoperative wound monitoring and care while minimizing the risk of maceration. Beyond the present case, the medial approach may also be applicable in other causes of high tibial nerve compression. Situations such as popliteal cysts, space-occupying lesions, traumatic or iatrogenic injuries, or anatomically deep popliteal fossa entrapment could similarly benefit from medial exposure, especially when a posterior approach is limited by scarring, deep tissue planes, or patient body habitus. Nevertheless, a larger series would be required to determine whether one approach yields consistently better outcomes. Fourth, the supine position, which is best suited for the medial approach, is particularly advantageous in obese patients to facilitate airway management, to reduce the risk of hemodynamic instability [16] related to abdominal or thoracic compression, and to reduce complications related to anesthesia when compared with prone positioning [17].

The concomitant popliteal vein thrombosis highlighted the potential risk of pulmonary embolism at decompression, suggesting that temporary inferior vena cava filter placement may be considered in selected cases.

This case illustrates an unusual cause of tibial nerve palsy resulting from spontaneous rupture and eventually necrosis of the popliteus muscle limited to its own fascial compartment. MRI proved essential for diagnosis, revealing both the rupture and the mass effect on the tibial nerve. A medial surgical approach executed in supine position allowed for safe and effective surgical decompression, consisting of excision of all necrotic tissue and of a release of the tibial nerve at the soleal arch. Neurological recovery was almost complete and persistent.

## 4. Conclusions

In practical terms, tibial nerve palsy associated with acute calf pain should prompt early consideration of popliteus muscle injury, especially when symptoms are recurrent or rapidly progressive. In patients presenting with sudden calf pain followed by sensory disturbance or motor deficit, clinicians should include popliteus rupture among the differential diagnoses and proceed to targeted MRI when initial clinical findings are unclear. Early suspicion is essential, as timely surgical decompression may prevent irreversible nerve damage. Although rare, this condition warrants a structured diagnostic approach based on three key steps: (1) recognizing the association between acute calf pain and evolving tibial neuropathy, (2) performing early imaging to identify isolated popliteus injuries, and (3) strongly considering prompt surgical decompression.

## Figures and Tables

**Table 1:** Reported cases of tibial nerve palsy secondary to popliteus muscle rupture (chronological order).

Patient [Ref.]	Trauma	Clinical Presentation	Imaging/EMG Findings	Treatment	Outcome
59 yo man [2]	Twisting of knee while playing tennis	Pain and swelling right calf; FHL, FDL, and TP: M0; ↓ sensation sole of foot	Venogram: obstruction; X-ray: degenerative changes; MRI: PM rupture and hematoma and TN compression; EMG: TN axonotmesis	Conservative	At 6 months: toe flexion recovered; no further details
41 yo female [12]	Running	Pain and swelling right calf; partial TN palsy; altered sensation sole of foot	X-ray: normal; MRI: PM rupture and fluid collection	Conservative	Not specified
58 yo female [5]	No trauma	Pain sole of left foot aggravated by ankle motion; FHL and FDL: M0; altered sensation sole of foot	CT and MRI: PM hypertrophy and high-signal lesion on T2-weighted; EMG: denervation FHL and FDL	Neurolysis posterior approach	At 12 months: No pain; Normal sensation sole; FHL and FDL: M4
59 yo man [14]	Fall from a horse	Biphasic progressive onset; complete TN palsy	X-ray: unremarkable; MRI: PM rupture and hemorrhage and edema and TN Compression	Conservative	At 24 months: toe flexion deficit; intrinsic palsy; protective sensation sole of the foot
57 yo man [4]	Tennis match/no trauma	Biphasic progressive onset; numbness of the sole of the foot; partial palsy; FHL and FDL: M3	X-ray: degenerative changes; MRI: PM enlargement and TN compression	Conservative	At 8 months: sole numbness FHL and FDL: M4
34 yo man [11]	Twisting injury	Biphasic progressive onset; immediate posterior knee pain; altered sensation sole of foot; toes flexion: M0	X-ray: unremarkable; MRI: PM rupture and necrosis and compression popliteal vein and TN	Neurolysis posterior approach	At 9 months: medial sensation recovered; FHL: M1; plantar flexion: M5
54 yo man [15]	Jump with knee hyperextension	Biphasic progressive onset; posterior knee pain; toe flexion weakness; loss sensation sole of foot; absent Achilles reflex	MRI: PM thickness, edema, and hemorrhage; EMG: TN palsy	Conservative	At 15 months: moderate improvement toe flexion, persistent pain, and sensory loss
57 yo man [13]	No trauma	Pain left calf; numbness sole of foot; FHL: M1; progressive worsening of symptoms	AngioCT: popliteal artery stenosis; MRI: PM edema and enlargement; EMG: TN palsy	Neurolysis posterior approach	Immediate pain relief after surgery. At 2 months: partial deficit; FHL still impaired

yo: years old; FHL: flexor hallucis longus; FDL: flexor digitorum longus; TP: tibialis posterior; PM: popliteus muscle; TN: tibial nerve. ↓—indicates a decrease.

## Data Availability

All data generated or analyzed during this study are included in this article. Further inquiries can be directed to the corresponding author.

## Abbreviations

The following abbreviations are used in this manuscript:

MRI	Magnetic resonance imaging (MRI)
TP	Tibialis posterior
FDL	Flexor digitorum longus
FHL	Flexor hallucis longus
PM	Popliteus muscle
TN	Tibial nerve
